# Microbial Community Responses and Nitrogen Cycling in the Nitrogen-Polluted Urban Shi River Revealed by Metagenomics

**DOI:** 10.3390/microorganisms13051007

**Published:** 2025-04-27

**Authors:** Ran Wang, Shang Yang, Wei Zhao

**Affiliations:** College of Heilongjiang River and Lake Chief, Heilongjiang University, Harbin 150080, China; wangran990924@163.com (R.W.); 18867352147@163.com (S.Y.)

**Keywords:** nitrogen pollution, Shi River, microbial community, *narG*, denitrification, metagenomics

## Abstract

Nitrogen pollution in urban rivers, exacerbated by rapid urbanization, poses a growing threat to water quality. Microbial communities are essential in mediating nitrogen cycling and mitigating pollution in these ecosystems. This study integrated three-year (2021–2023) water quality monitoring with metagenomic sequencing to investigate microbial community dynamics, nitrogen cycling processes, and their responses to nitrogen pollution in the Shi River, Qinhuangdao, China. Nitrogen pollution was predominantly derived from industrial discharges from enterprises in the Shi River Reservoir upstream (e.g., coolant and chemical effluents), agricultural runoff, untreated domestic sewage (particularly from catering and waste in Pantao Valley), and livestock farming effluents. Total nitrogen (TN) concentrations ranged from 2.22 to 6.44 mg/L, exceeding China’s Class V water standard (2.0 mg/L, GB 3838-2002), with the highest level at the urbanized W4 site (6.44 mg/L). Nitrate nitrogen (NO_3_-N) accounted for 60–80% of TN. Metagenomic analysis revealed Fragilaria, Microcystis, and Flavobacterium thriving (up to 15% relative abundance) under nitrogen stress, with nitrogen metabolism genes (*narG*, *nifH*, *nirK*) enriched at polluted sites (W2, W4), *narG* reaching 26% at W1. Dissolved oxygen positively correlated with nitrate reductase gene abundance, while ammonia nitrogen inhibited it. Burkholderiales and Limnohabitans dominated denitrification, offering insights into sustainable urban river management.

## 1. Introduction

Rapid urbanization since the 20th century has significantly increased nitrogen pollution in urban rivers, disrupting ecosystems worldwide [1]. This pollution, primarily from agricultural runoff, industrial effluents, and untreated sewage, elevates nutrient levels, particularly nitrogen compounds, in river waters [2,3]. Such shifts lead to excessive nutrient loads, altering surface morphology, hydrology, and habitats, thereby threatening freshwater ecosystem integrity [4]. In urban rivers, nitrogen pollution often triggers eutrophication, promotes harmful algal blooms, and reduces dissolved oxygen, severely impacting aquatic life and water quality [5]. These changes challenge urban sustainability by affecting biodiversity and ecosystem services like water purification and flood regulation.

As vital components of urban ecosystems, rivers such as the Shi River in Qinhuangdao City, China, reflect regional environmental health and support resident well-being [6]. Spanning 79.6 km with a 618 km^2^ drainage area, the Shi River serves as a critical water source and ecological corridor. Recent investigations reveal rising nitrogen concentrations, posing a growing threat to its water quality. However, comprehensive studies on its microbial community structure and response to nitrogen pollution remain limited despite their importance for ecological health [7].

Recent research highlights how urbanization alters microbial nitrogen cycling in rivers. Urban rivers exhibit higher nitrogen accumulation (e.g., 4.51–8.79 mg/L total nitrogen) compared to suburban systems, driven by reduced abundances of functional genes for nitrification, denitrification, and anammox, particularly in summer [8]. Anammox, a key nitrogen removal pathway, achieves rates up to 3.64 μmol N g^−1^ h^−1^ in spring urban sediments but declines in summer due to hypoxia. Moreover, urban rivers show lower microbial diversity, with dominance by anaerobic respiring bacteria (e.g., Firmicutes at 40.9% in spring), contrasting with suburban rivers rich in Proteobacteria [9]. This reduced diversity correlates negatively with nitrogen removal efficiency, exacerbating pollution [10].

This study aims to monitor the Shi River’s physicochemical parameters (pH, dissolved oxygen, ammonia nitrogen, total phosphorus, and total nitrogen) over three years, alongside metagenomic analysis of microbial communities across river sections in October 2023. By correlating microbial composition with environmental factors, we seek to elucidate microbial roles in nitrogen cycling and pollution mitigation, providing insights for sustainable management in Qinhuangdao and similar urban regions [11].

## 2. Materials and Methods

### 2.1. Study Area

**Figure 1:**
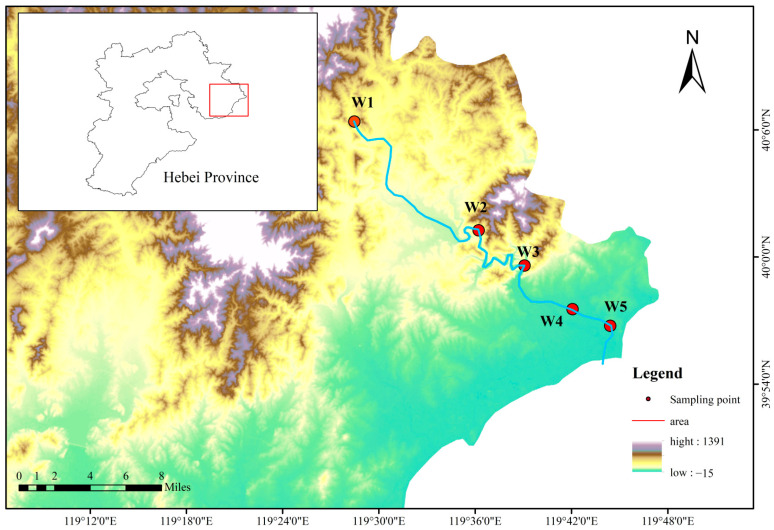
Position of the research area and sampling sites.

The Shi River, originating southeast of Liujialing in Qinglong County, Qinhuangdao City, Hebei Province, China, flows 79.6 km through Shanhaiguan District into the Bohai Sea, with a 618 km^2^ drainage area. This river, transitioning from a seasonal to a perennial flow after the Dafengkou Reservoir’s construction, has a mean annual discharge of 1.286 × 10^8^ m^3^. It traverses mountainous upstream regions with good vegetation cover and urbanized downstream areas, receiving pollutants primarily from agricultural runoff and urban sewage. Five monitoring sections were established along its mainstream: Yingfang Village (W1) monitors upstream runoff; Pantaoyu (W2) tracks midstream water quality; the dam (W3) near the Shi River Reservoir assesses reservoir outflow; the railway bridge (W4) evaluates urban water quality; and the Shi River mouth (W5) monitors quality before entering the sea. Sampling site locations and additional hydrological details are provided in Figure 1.

### 2.2. Sample Collection and Analysis

#### 2.2.1. Testing of Water Quality Physical and Chemical Indicators

Water samples were collected monthly from January to October 2021–2023 at five sites (W1–W5) at a 0.5 m depth using sterile containers, following the Water Quality Sampling Technical Guidance (HJ 494—2009) [12]. Water samples were analyzed for a comprehensive suite of physicochemical parameters to evaluate water quality. The parameters assessed included pH, total phosphorus (TP), total nitrogen (TN), dissolved oxygen (DO), nitrate nitrogen (NO_3_^−^-N), ammonia nitrogen (NH_3_-N), and chemical oxygen demand (COD). Each parameter was measured in strict accordance with the respective national standard methodologies: pH was determined following GB/T 6920-1986 [13], TP via GB 11893-89 [14], TN using HJ 636-2012 [15], DO according to GB 7489-87 [16], NO_3_^−^-N by HJ/T 84-2001 [17], NH_3_-N as per HJ 536-2009 [18], and COD following GB/T 32208-2015 [19]. These standardized methods ensure the reliability and comparability of the data collected, facilitating a robust assessment of water quality.

#### 2.2.2. DNA Extraction

Surface water samples were collected from five sites (W1–W5) along the Shi River in October 2023 at a depth of 0.5 m. For each site, approximately 2 L of water were filtered through 0.22 μm pore size membrane filters (Millipore, Billerica, MA, USA) to capture microbial biomass. The filters were immediately stored at −8 °C until DNA extraction.

Genomic DNA was extracted using the E.Z.N.A.^®^ Soil DNA Kit (Omega Bio-Tek, Norcross, GA, USA) following the manufacturer’s instructions. DNA quality and integrity were assessed via 1% agarose gel electrophoresis. Subsequently, DNA was fragmented using a Covaris M220 ultrasonicator (Gene Company, Shanghai, China) to generate ~350 bp fragments, which were then used to construct paired-end (PE) sequencing libraries using the NEXTFLEX^®^ Rapid DNA-Seq Kit (Bioo Scientific, Austin, TA, USA).

High-throughput metagenomic sequencing was performed on the Illumina NovaSeq 6000 platform (Illumina, San Diego, CA, USA).

#### 2.2.3. Methods of Analysis

Raw shotgun sequencing reads generated from the microbial samples were first subjected to preprocessing to ensure data quality for downstream analyses. Adapter sequences at the 3′ and 5′ ends of the reads were trimmed using fastp (version 0.20.0, https://github.com/OpenGene/fastp), a fast and versatile tool designed for quality control of high-throughput sequencing data. Trimming was performed with a Phred quality score threshold of Q20 (99% base call accuracy), and reads were filtered to remove those with average quality below this cutoff, more than 5% ambiguous bases (N), or lengths shorter than 50 bp post-trimming. This stringent filtering enhances assembly accuracy by reducing noise from low-quality sequences and short fragments, a critical step validated in metagenomic studies for improving contig integrity.

The preprocessed high-quality reads were then assembled de novo into contigs using MEGAHIT (version 1.1.2, https://github.com/voutcn/megahit), an assembler optimized for complex metagenomic datasets. MEGAHIT employs a succinct de Bruijn graph approach, with k-mer sizes incrementally ranging from 21 to 141 (step size of 10), allowing efficient assembly of both abundant and rare microbial genomes. Contigs shorter than 300 bp were discarded to minimize assembly artifacts and focus on biologically significant sequences, yielding a robust set of genomic fragments for gene prediction. 

Open reading frames (ORFs) within these contigs were identified using Prodigal (version 2.6.3, https://github.com/hyattpd/Prodigal), a prokaryotic gene-finding tool tailored for metagenomic applications. Running in metagenomic mode (-p meta), Prodigal predicted ORFs without prior taxonomic assumptions, selecting those with nucleotide lengths ≥ 100 bp to ensure coding potential. These ORFs were translated into amino acid sequences using the standard genetic code, preparing them for functional annotation.

To construct a non-redundant gene catalog, predicted gene sequences from all samples were clustered using CD-HIT (version 4.7, https://github.com/weizhongli/cdhit). Clustering was performed at 90% nucleotide identity and 90% coverage. Thresholds were chosen to balance gene diversity with redundancy reduction. The longest gene in each cluster was selected as the representative sequence, forming a consolidated gene set for abundance and functional profiling.

Gene abundance was quantified by mapping high-quality reads from each sample back to the non-redundant gene set using SOAPaligner (version soap2.21, https://github.com/ShujiaHuang/SOAPaligner). Alignments were conducted with a 95% identity threshold, allowing up to two mismatches, and only uniquely mapped reads were used to calculate abundance, normalized as reads per kilobase per million (RPKM) to account for gene length and sequencing depth variations. This approach ensures an accurate representation of gene prevalence across samples.

Functional annotation was performed by aligning the amino acid sequences of the non-redundant gene set against the KEGG database using Diamond (version 2.0.13, https://github.com/bbuchfink/diamond). Diamond’s BLASTP algorithm was applied with an e-value threshold of 1 × 10^−5^ and a bit-score cutoff of 60, assigning KEGG Orthology (KO) terms, pathways, and modules to each gene. The abundance of each functional category was then calculated by summing the RPKM-normalized abundances of all genes mapped to that category, providing a comprehensive view of the microbial community’s metabolic potential.

### 2.3. Biodiversity Analysis

Alpha diversity (Shannon, Chao1, ACE, Simpson) was analyzed using MOTHUR. Venn diagrams and correlation heatmaps were generated with MOTHUR, while genus-level community structure was visualized using Circos. Canonical Correspondence Analysis (CCA) was performed using the R vegan package (version 2.4.3) to assess relationships between microbial communities and environmental factors. To deal with multicollinearity, variance inflation factor (VIF) analyses were used, retaining only those variables with a VIF < 10; spatial autocorrelation between sampling points was assessed by Mantel’s test, which did not reveal significant spatial structure (*p* > 0.05).

### 2.4. Correlation Network Analysis

Networkx (version 1.11) in Python calculated network metrics (e.g., node degree, network diameter, average shortest path) and centrality attributes (Degree, Closeness, Betweenness) to investigate microbial interactions and community structure across species and samples, visualized with Gephi 0.9.2 [20].

### 2.5. Analysis of Species Contribution to Function

To investigate the relationships between microbial taxa and their associated functional capacities, we performed correlation-based analyses between species abundance and functional abundance using Python 3.1.1 and relevant scientific libraries (e.g., NumPy 2.2.0, SciPy 1.15.1, pandas2.2.3). Both taxonomic and functional profiles were normalized to relative abundance prior to analysis.

Pairwise correlations between the relative abundance of each species and each predicted functional category were computed using Spearman’s rank correlation coefficient, which is robust to non-parametric distributions common in microbial community data [21]. This approach enabled the identification of taxa that are significantly associated with specific metabolic functions or pathways, thereby allowing inference of potential functional contributions of individual species to the community-level functional landscape [22].

To further elucidate the ecological role of dominant taxa, we also examined function-to-species mappings, identifying core species that disproportionately contribute to key metabolic pathways. This dual-directional analysis framework provides insights into the functional redundancy and specialization within microbial communities.

## 3. Results

### 3.1. Shi River Water Quality Characteristics Analysis

#### 3.1.1. 2021–2023 Water Quality Analysis

**Figure 2:**
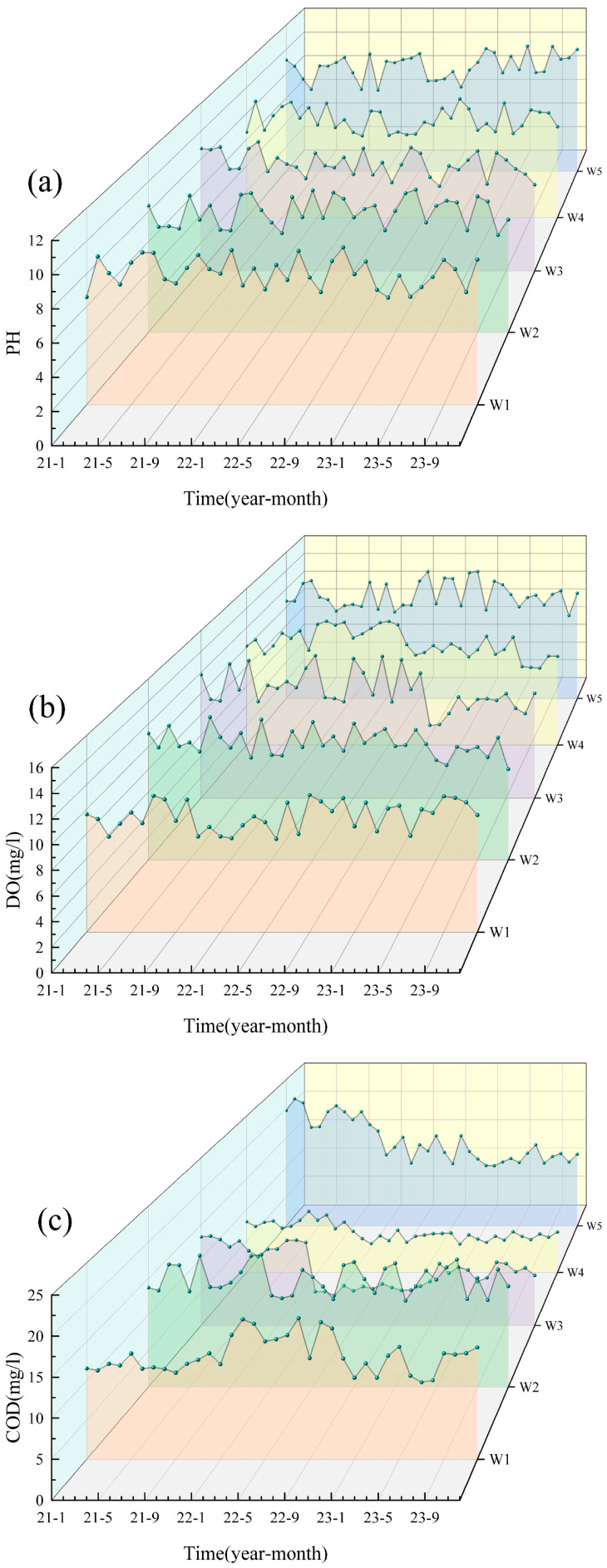

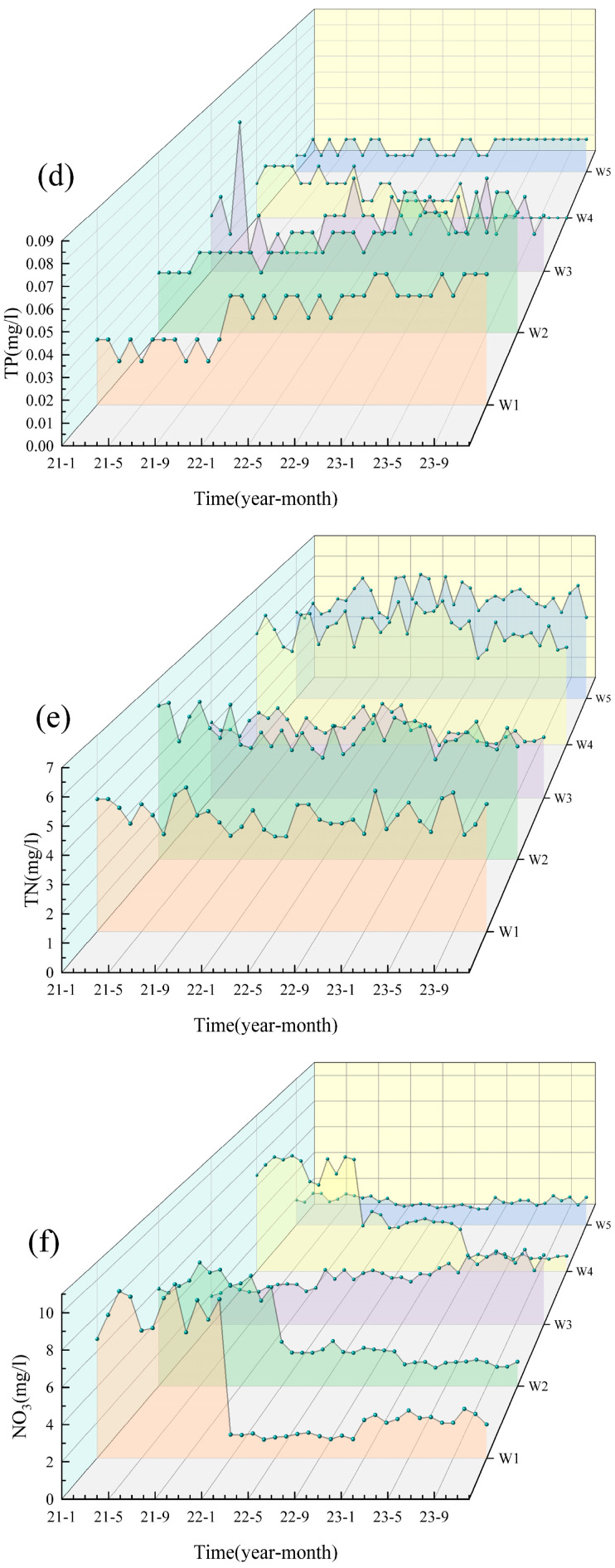

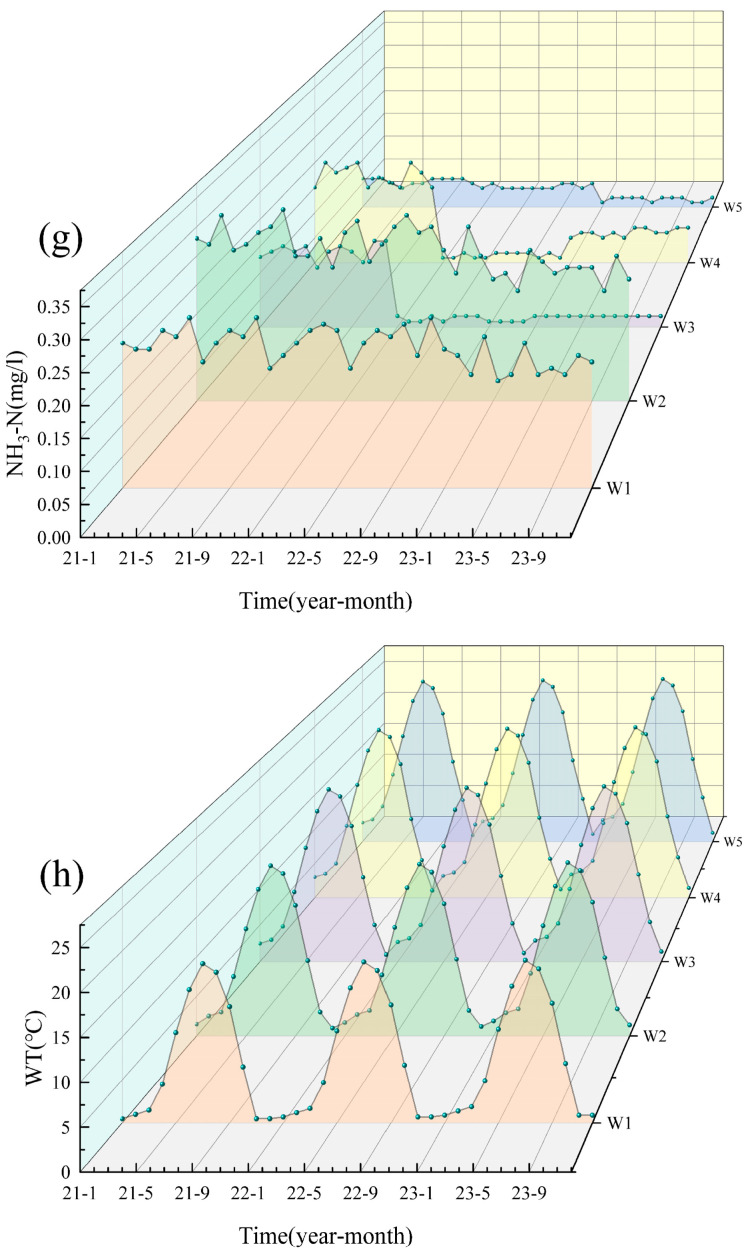
Analysis of the water quality from 2021 to 2023. (**a**) pH; (**b**) DO; (**c**) COD; (**d**) TP; (**e**) TN; (**f**) NO_3_; (**g**) NH_3_-N; (**h**) WT.

Based on the water quality data for the five cross-sections (W1–W5) of the Shi River from 2021–2023, the water quality indicators were generally stable from year to year and did not show a continuous deteriorating trend (Figure 2). Most pollutants (e.g., NH_3_-N, TP) peaked in 2022 and then declined in 2023, indicating an improvement in water quality. Particularly in summer (June–August), concentrations of nitrogen and phosphorus pollutants increased significantly due to high temperatures and rainfall during the flood season. TN, NO_3_-N and NH_3_-N peaked in summer, with the highest concentration of TN reaching W4 (6.44 mg/L) in July 2022, which exceeded the national Class V standard (2.0 mg/L). In contrast, water quality improved during the winter months (December–February) when pollutant concentrations generally decreased due to reduced water flow and cooler conditions. DO decreased in summer due to the decomposition of organic matter and increased microbial activity. It recovered in winter due to the increased reoxygenation capacity of the water body at low temperatures. WT showed significant seasonal fluctuations, peaking in summer (e.g., 23–27 °C at W4 and W5 in July 2022) and dropping to 0–2 °C in winter (e.g., January 2021 and 2023) across all sites.

In terms of spatial distribution, the upstream section (W1) and the reservoir section (W3) had better water quality with lower nitrogen and phosphorus concentrations, while the midstream urban section (W4) had significantly higher pollutant concentrations, especially TN, NH_3_-N and TP, showing the superimposed effects of urban wastewater and agricultural surface sources [23]. Water quality in the downstream section (W5) recovered but was still affected by upstream pollution inputs, and pollutant concentrations did not fully recover [24]. The distribution of NO_3_-N varied with the direction of water flow, indicating that the self-purification of the river contributed to the reduction in nitrogen pollution [25]. Overall, the water quality of the Shi River showed significant variation in different seasons and at different cross-sections. The seasonal and spatial differences reflect the dynamic changes in water quality and the different sources of pollution.

#### 3.1.2. Nitrogen Pollution Analysis

**Figure 3:**
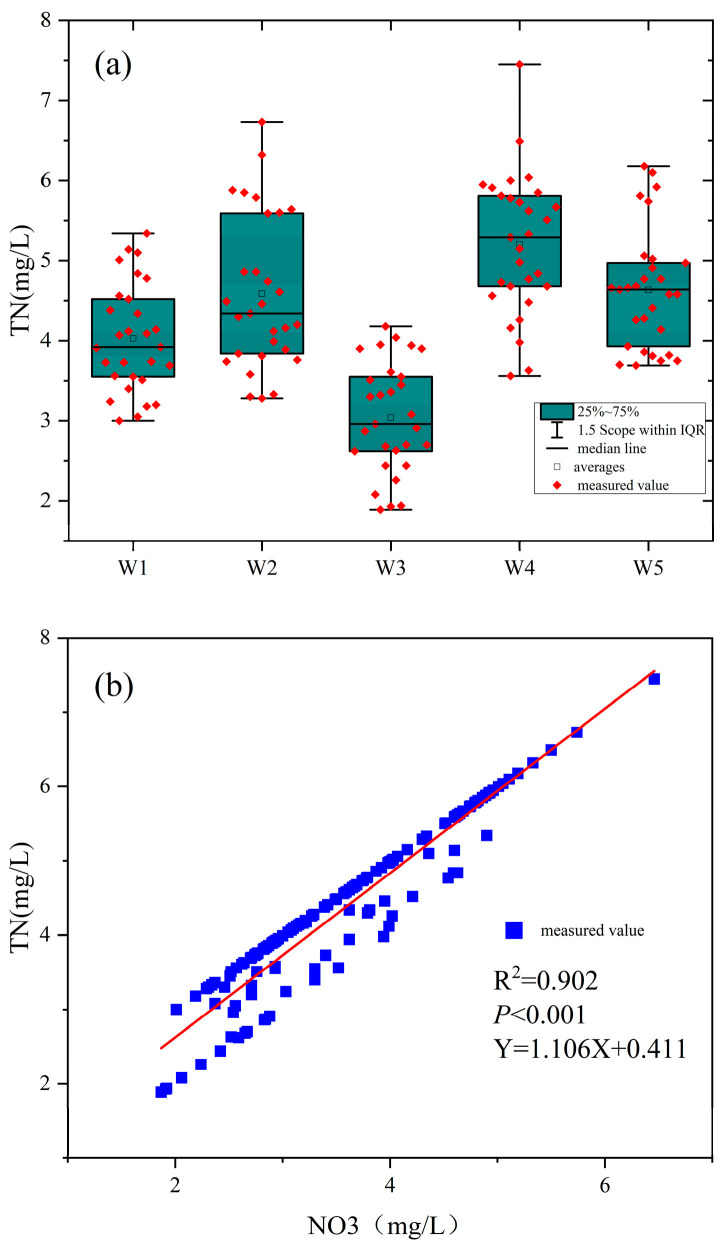
Analysis of nitrogen pollution. (**a**) total nitrogen pollution; (**b**) Nitrate contamination.

Further analysis of nitrogen pollution across the five sections (W1–W5) revealed distinct spatial and temporal patterns in TN and NO_3_-N concentrations (Figure 3). Figure 3a presents boxplots of TN concentrations over 2021–2023, showing medians, interquartile ranges (IQR), and outliers for each site. W4 exhibited the highest median TN (5.8 mg/L, IQR: 4.5–6.4), followed by W1 (4.2 mg/L, IQR: 3.8–4.8), W2 (3.5 mg/L, IQR: 2.8–4.2), W5 (2.9 mg/L, IQR: 2.5–3.3), and W3 (2.4 mg/L, IQR: 2.2–2.7). W4 showed the largest fluctuation, with outliers reaching 6.44 mg/L in July 2022, while W2 also displayed notable variability (IQR: 1.4 mg/L). In contrast, W3 was the most stable (IQR: 0.5 mg/L), likely due to a single nitrogen source and stricter pollution control. W1, near rural areas, and W4, at the urban periphery, exhibited severe nitrogen pollution, with NO_3_-N contributing approximately 70% of TN at W4, attributed to low-lying topography, poor drainage, and direct sewage discharge [26]. The annual mean values of NO_3_-N/TN ratios from 2021–2023 were 68.2%, 51.3%, and 55.8%, respectively, with small inter-seasonal variations (60.1% in spring ~59.5% in winter); spatially, the highest ratio was found in W3 of the sampling sites (78.3%), and the lowest in W5 (39.8%). Statistical tests showed significant differences between sampling sites (F = 45.67, *p* < 0.001) and non-significant differences between seasons (e.g., W1, H = 2.34, *p* = 0.505).

Figure 3b illustrates a scatter plot of TN versus NO_3_-N concentrations across all samples, revealing a strong positive correlation (R^2^ = 0.902, *p* < 0.001, Y = 1.106X − 0.411). NO_3_-N, as the dominant form of nitrogen pollution, accounted for 60–80% of TN across sites, a pattern linked to high DO levels (6.8–9.5 mg/L) that favor nitrate production and stabilization [27,28]. Elevated TN and NO_3_-N levels, particularly at W4, increase the risk of eutrophication in the Shi River, potentially leading to algal blooms, DO depletion, biodiversity loss, and toxicity to sensitive aquatic species [29,30,31].

### 3.2. Shi River Biodiversity Analysis

#### 3.2.1. Alpha Diversity Analysis

Alpha diversity indices (Shannon, Simpson, Chao1, ACE, Shannon evenness) of microbial communities from five Shi River sections (W1–W5) were calculated for 2023 (Table 1). Table 1 presents the mean values of diversity indices. Shannon indices ranged from 4.64 to 5.25, peaking at W5 (5.25) and lowest at W1 (4.64), with intermediate values at W4 (5.17), W2 (5.03), and W3 (4.88). Simpson indices, reflecting dominance, ranged from 0.0143 (W5) to 0.0415 (W1), indicating higher evenness at W5. Shannon evenness followed a similar trend, increasing from 0.5361 (W1) to 0.6052 (W5). Chao1 and ACE, both measures of species richness, were highest at W4 (5986) and W5 (5881) and lowest at W2 (5119), with W1 (5776) and W3 (5702) showing intermediate richness.

#### 3.2.2. Bacterial Community Structure Analysis

**Figure 4:**
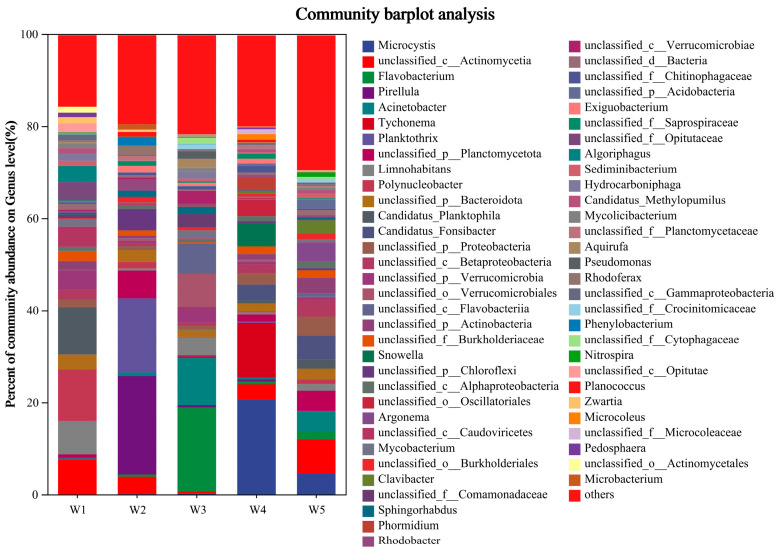
Analysis of microbial community structure at genus level.

The distribution of microbial structure at the genus level in the Shi River is illustrated in Figure 4 (microbial community structure at genus level, abundance greater than 1%). The dominant genera in all samples included *Fragilaria*, *unclassified_c__Actinomycetia*, *Microcystis*, *Nitzschia*, *Flavobacterium*, and *Phaeodactylum*. *Fragilaria* is a type of diatom adapted to silicon-rich environments and is generally indicative of higher silicon content in water bodies. *Unclassified_c__Actinomycetia* refers to bacterial sequences that have been identified as belonging to the class Actinomycetia, which encompasses a variety of Gram-positive, high-GC content bacteria that play important ecological and applied roles in soil decomposition, environmental nutrient cycling, and the biosynthesis of secondary metabolites such as antibiotics. *Microcystis* are nitrogen-fixing cyanobacteria that typically thrive in eutrophic water bodies abundant in nitrogen and phosphorus. *Nitzschia* is another diatom that adapts to water bodies with higher nitrogen content and plays a role in nutrient cycling [32]. *Flavobacterium* is a bacterium associated with organic matter degradation and nitrogen metabolism that contributes to biogeochemical processes [33]. *Phaeodactylum*, a diatom commonly found in eutrophic waters, plays an important role in nutrient cycling in the water [34].

The following dominant genera were identified at each sample site: *Planktothrix, Candidatus_Planktophila*, *unclassified_c__Actinomycetia*, *Limnohabitans* and *unclassified_p__Verrucomicrobia* at W1; *Pirellula, Planktothrix*, *unclassified_p__Chloroflexi*, *unclassified_p__Planctomycetota* and *unclassified_c__Actinomycetia* at W2. In W3, the genera present include *Flavobacterium*, *Acinetobacter*, *unclassified_o__Verrucomicrobiales*, *unclassified_c__Flavobacteriia* and *Limnohabitans*. W4 is characterised by the dominance of *Microcystis*, *Tychonema*, *Snowella*, *unclassified_o__Oscillatoriales* and *Candidatus_Fonsibacter*. The most prevalent genera in W5 are *unclassified_c__Actinomycetia*, *Candidatus_Fonsibacter*, *Microcystis*, *unclassified_p__Planctomycetota* and *Acinetobacter*.

#### 3.2.3. Bacteria Nitrogen Metabolism Functional Gene Analysis

**Figure 5:**
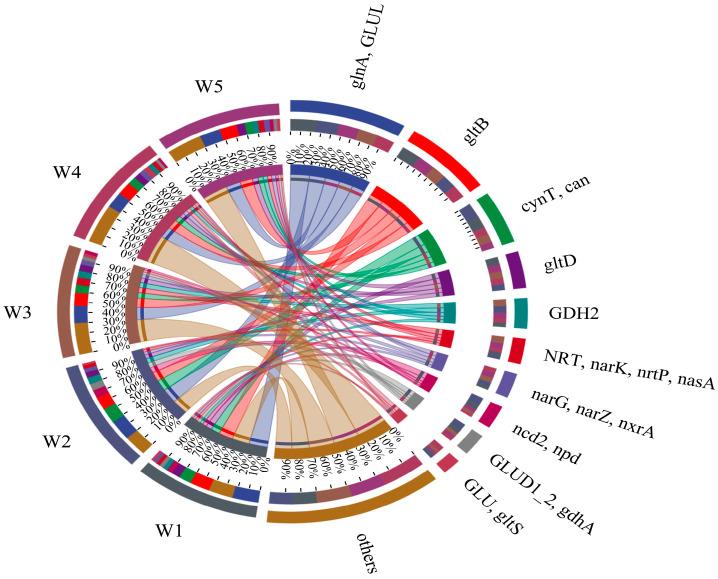
Nitrogen metabolism functional gene analysis.

Nitrogen metabolism functional genes across five Shi River sections (W1–W5) were analyzed using high-throughput sequencing from 2021–2023 (Figure 5). Figure 5 presents a Circos plot illustrating the relative abundances of key nitrogen metabolism genes (>1% abundance) and their co-occurrence patterns across sampling sites. Dominant genes included *narG*, *narZ*, *nxrA* (encoding nitrate reductase, involved in denitrification), *glnA*, *GLUL* (encoding glutamine synthetase, involved in nitrogen assimilation), *gdhA*, *GLUD1–2* (encoding glutamate dehydrogenase, involved in nitrogen assimilation), *nirS* (encoding nitrite reductase, involved in denitrification), and *narK*, *nrtP*, *nasA* (encoding nitrite reductase, involved in denitrification).

The distribution of gene abundances showed distinct patterns across sampling sites. *narG*, *narZ*, *nxrA* (nitrate reductase) was highest in W1 (26.0%) and W2 (21.7%) and lowest in W4 (17.2%). The DO concentration at W4 was consistently high throughout the study period, ranging from 7.85 to 12.66 mg/L. This elevated DO level is likely to have suppressed denitrification, as the process typically occurs under low-oxygen or anaerobic conditions. *glnA*, *GLUL* (glutamine synthetase) had the highest abundance in W1 (25%) and W5 (22%), while it was largely consistent in the other three regions (18%), suggesting that nitrogen assimilation is enhanced in areas with high nitrogen inputs. *gdhA*, *GLUD1–2* (glutamate dehydrogenase) showed the highest expression in W2 (31%), followed by W3 (23.0%), and the lowest in W5 (13%), possibly due to varying nitrogen and oxygen concentrations. K00266 (nirS, nitrite reductase) was highest in W1 (23%) and W5 (22%) and lowest in W2 (17%), indicating active denitrification in nitrogen-rich zones [35]. Finally, *narK*, *nrtP*, *nasA* (nitrite reductase) had the highest expression in W3 (32%) and the lowest in W2 (15%) and W1 (14%), which may reflect reduced denitrification activity in estuaries prior to discharge [36].

### 3.3. Shi River Microbial Community Response to Nitrogen Pollution

#### 3.3.1. Effects of Water Quality Factors on Bacterial Communities

**Figure 6:**
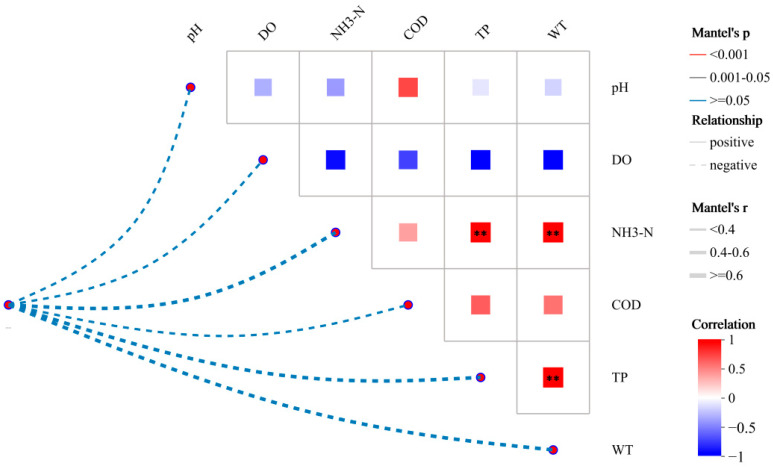
Environmental factor correlation analysis. (**: 0.001 < *p* ≤ 0.01).

The effects of environmental factors on bacterial communities in the Shi River were evaluated using Mantel tests and correlation analysis (Figure 6). Figure 6 illustrates the relationships between pH, DO, NH_3_-N, COD, TN, TP, WT, and bacterial community variation. As NO_3_-N makes up a significant proportion of TN and the two have a strong positive correlation (R^2^ = 0.902), TN was used as a representative measure of nitrogen pollution in subsequent environmental factor correlation analyses. This approach was adopted to avoid problems of multicollinearity, which could affect the results of the analysis [37].

Mantel tests identified TN as the primary driver of bacterial community changes (r ≈ 0.6, *p* < 0.001), followed by NH_3_-N (r ≈ 0.4, *p* < 0.05), WT (r ≈ 0.35, *p* < 0.05), TP (r ≈ 0.3, *p* < 0.05), pH (r ≈ 0.25, *p* > 0.05), DO (r ≈ 0.2, *p* > 0.05), and COD (r ≈ 0.15, *p* > 0.05). The correlation heatmap revealed significant negative correlations between TN and both pH (r ≈ −0.5, *p* < 0.001) and DO (r ≈ −0.6, *p* < 0.001), a strong positive correlation between NH3-N and TN (r ≈ 0.8, *p* < 0.001), and a negative correlation between DO and NH3-N (r ≈ −0.4, *p* < 0.05). The high correlation between TN and NH_3_-N was particularly evident at W4, where TN (5.8 mg/L) and NH3-N (1.2 mg/L) were highest, and DO was lowest (6.8 mg/L).

#### 3.3.2. Nitrogen Metabolism Functional Genes

**Figure 7:**
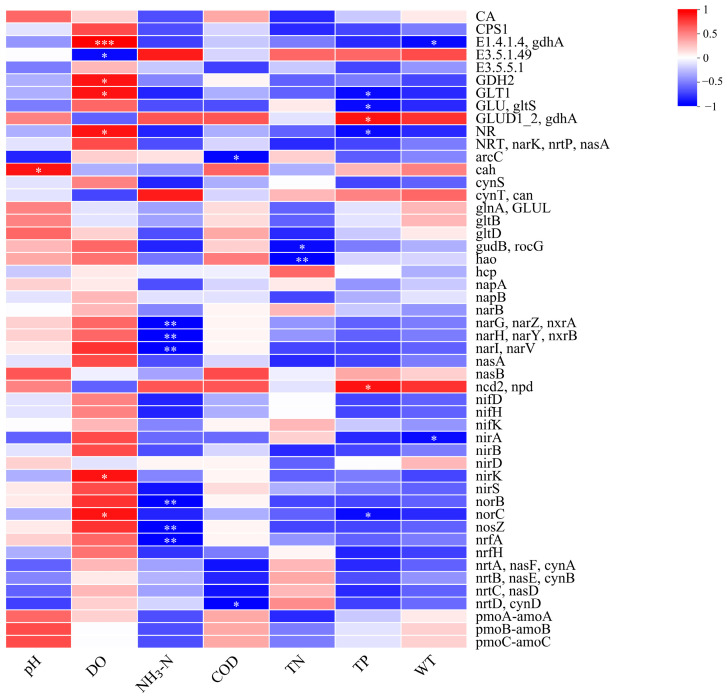
Correlation analysis of environmental factors and functional enzymes of nitrogen metabolism. (*: 0.01 < *p* ≤ 0.05, **: 0.001 < *p* ≤ 0.01, ***: *p* ≤ 0.001).

The correlation between environmental factors and nitrogen metabolism functional genes in the Shi River was assessed using correlation analysis (Figure 7). Figure 7 presents a heatmap of correlations between pH, DO, NH_3_-N, COD, TN, TP, WT, and nitrogen metabolism genes. DO showed significant positive correlations with several genes, including *narG*, *narZ*, *nxrA* (encoding nitrate reductase, r ≈ 0.6, *p* < 0.01), *nirK* (encoding nitrite reductase, r ≈ 0.5, *p* < 0.01), and *narL*, *narV* (encoding nitrate reductase, r ≈ 0.4, *p* < 0.05). NH_3_-N exhibited significant negative correlations with these genes (*narG*, *narZ*, *nxrA*: r ≈ −0.55, *p* < 0.01; *narH*, *narY*, *nxrB*: r ≈ −0.45, *p* < 0.01; *narI*, *narV*: r ≈ −0.4, *p* < 0.05). *nifH* (encoding nitrogenase) was negatively correlated with DO (r ≈ −0.5, *p* < 0.01) and positively correlated with NH_3_-N (r ≈ 0.4, *p* < 0.05). *argF* (encoding an ammonia metabolism enzyme) showed a positive correlation with NH_3_-N (r ≈ 0.6, *p* < 0.01). TN was positively correlated with *nirS* (encoding nitrite reductase, r ≈ 0.5, *p* < 0.01), particularly evident at W4 (TN: 5.8 mg/L, DO: 6.8 mg/L).

#### 3.3.3. Bacterial Symbiotic Pattern Analysis

**Figure 8:**
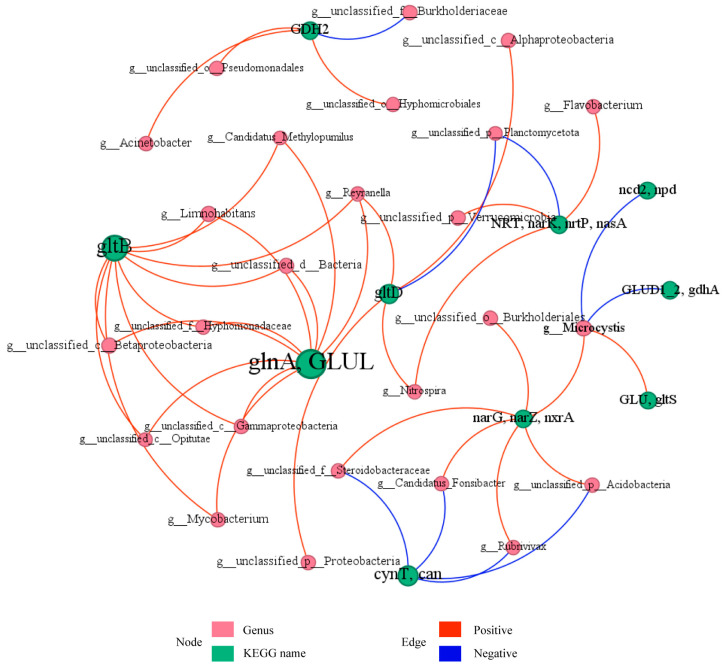
Network analysis of relationships between microbial communities and functional genes for nitrogen metabolism.

Bipartite co-occurrence network of microbial genera and nitrogen metabolism genes in the Shi River microbial community (Figure 8), comprising 35 nodes (20 microbial genera, pink; 15 genes, green) and 45 edges (*p* < 0.05). Node size reflects abundance, with dominant nodes including *g__Microcystis* (abundance = 2376), *g__Limnohabitans* (abundance = 1820), *glnA*, *GLUL* (abundance = 24,342), and *gltB* (abundance = 18,708). Solid red edges (70%) indicate positive correlations and dashed blue edges (30%) represent negative correlations, with thickness proportional to |r|. Key interactions include *g__Microcystis* with *narG*, *narZ*, *nxrA* (r = 0.9, *p* < 0.05) and *GLUD1_2*, *gdhA* (r = −0.9, *p* < 0.05), *g__Limnohabitans* with *glnA*, *GLUL* (r = 0.9, *p* < 0.05), and *g__Reyranella* with *glnA*, *GLUL* (r = 1.0, *p* < 0.001). Modularity analysis (Q ≈ 0.60) identified three modules: Module 1 (12 nodes) centers on *glnA*, *GLUL* and *gltB*, with strong positive correlations (average r ≈ 0.95, *p* < 0.05) to *g__Limnohabitans* and *g__Reyranella*; Module 2 (10 nodes) is led by *narG*, *narZ*, *nxrA*, showing mixed interactions with Module 1 (e.g., *g__Microcystis* with *glnA*, *GLUL*, r ≈ −0.9, *p* < 0.05); Module 3 (8 nodes) includes *NRT*, *narK*, *nrtP*, *nasA*, positively correlated with *g__unclassified_p__Verrucomicrobia* and *g__Nitrospira* (r ≈ 0.9, *p* < 0.05). The network highlights *glnA* and *GLUL* as the central hub, with synergistic interactions predominating.

#### 3.3.4. Functional Gene Analysis of Shi River Bacteria

**Figure 9:**
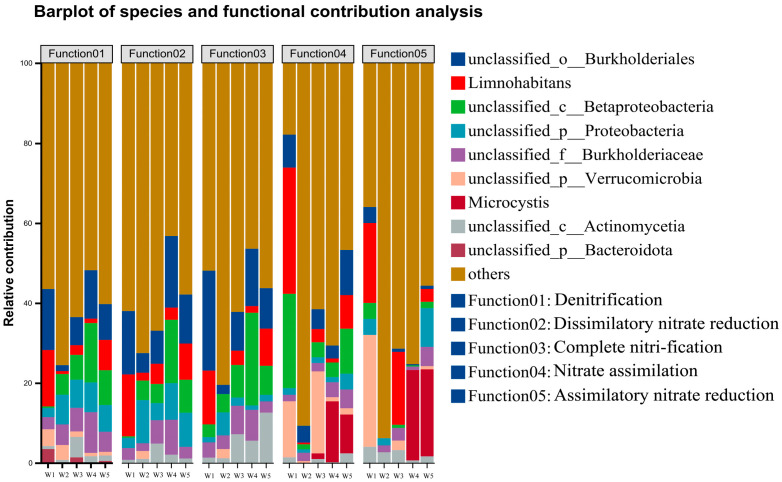
Predictions of species contributions to function.

The contributions of microbial genera to nitrogen metabolism pathways across five Shi River sections (W1–W5) were analyzed using high-throughput sequencing (Figure 9). Figure 9 presents stacked bar charts of relative contributions from dominant genera (>1% abundance) to five nitrogen metabolism pathways (M00529: denitrification, M00530: dissimilatory nitrate reduction, M00804: complete nitrification, M00615: nitrate assimilation, M00531: assimilatory nitrate reduction). In M00529 (denitrification), *unclassified_o_Burkholderiales* contributed the most at W4 (35%), followed by *Limnohabitans* at W2 (25%). M00530 (dissimilatory nitrate reduction) was dominated by *unclassified_c_Betaproteobacteria* at W4 (30%), with *unclassified_o_Burkholderiales* contributing ~20% at W1. For M00804 (complete nitrification), *Limnohabitans* contributed 20% at W3, and *unclassified_c_Betaproteobacteria* contributed ~15% at W5. In M00615 (nitrate assimilation), *unclassified_p_Verrucomicrobia* dominated at W4 (40%), while Microcystis contributed 30% at W3. M00531 (assimilatory nitrate reduction) showed high variability across sites, with *unclassified_p_Actinomycetia* contributing 25% at W1 and Microcystis contributing 20% at W5.

## 4. Discussion

### 4.1. Nitrogen Pollution Drivers and Spatial Patterns

Nitrogen pollution in the Shi River exhibited significant spatial heterogeneity, closely linked to the geographical locations and pollution sources of the sampling sites. WT at W1 was consistently lower due to higher elevation, while W4 and W5 exhibited higher WT, correlating with elevated TN and microbial activity at these sites. W4 recorded the highest TN concentration (5.8 mg/L, peaking at 6.44 mg/L), far exceeding China’s Class V surface water standard(GB 3838-2002) [38]. TN concentrations at W4 decreased from 6.44 mg/L in July 2022 to 4.5 mg/L in July 2023, likely due to reduced precipitation (from 700 mm in 2022 to 550 mm in 2023) and cooler annual mean temperatures (from 11.0 °C in 2022 to 10.8 °C in 2023), while W1 and W3 showed lower TN levels (2.22 mg/L and 2.4 mg/L, respectively). Located in an urban area with low-lying topography and poor drainage, W4 likely experienced direct sewage discharge [39], contributing to its elevated TN (2.1 mg/L). More specifically, this sewage likely originates from untreated wastewater in Shanhaiguan District, where W4’s proximity to the railway bridge captures urban discharges from residential and commercial activities. Additionally, W2, with a moderate TN of 3.5 mg/L, reflects a mix of agricultural runoff and emerging urban influences from nearby towns like Pantaoyu, alongside potential industrial discharges from enterprises in Shimen Village and Zhucaoying Town, which release nitrogen-containing wastewater such as cooling fluids. Additionally, NO_3_-N accounted for 60–80% of TN, correlating with high DO levels (6.8–9.5 mg/L), suggesting that high oxygen conditions favored the oxidation of ammonia to nitrate. However, W4’s lowest DO (6.8 mg/L) may have limited nitrification, leading to NH_3_-N accumulation (1.2 mg/L). In contrast, W1, situated in the upstream mountainous region with good vegetation cover, was primarily influenced by agricultural runoff [40], resulting in lower TN and reflecting reduced pollution inputs. W3, near the reservoir, exhibited the lowest TN, likely due to stricter pollution control and the reservoir’s role in nitrogen sedimentation and dilution [41]. At W5, located at the Shi River mouth, TN levels are likely elevated due to the cumulative effects of upstream pollution, compounded by additional urban sewage inputs from Shanhaiguan District, though tidal mixing may partially dilute nitrogen concentrations. These findings indicate that urbanization and land use patterns are the primary drivers of nitrogen pollution in the Shi River, consistent with studies highlighting the impact of urban sewage and agricultural runoff on riverine nitrogen pollution [42].

### 4.2. Spatial Patterns of Microbial Alpha Diversity

The spatial variation in microbial alpha diversity along the Shi River reflects the influence of environmental heterogeneity and nutrient dynamics. The Shannon diversity index increased from upstream (W1, 4.64) to downstream (W5, 5.25), while the Simpson index decreased, indicating reduced dominance and greater evenness in downstream communities. Higher Shannon evenness and richness indices (Chao1 and ACE) at W4 and W5 suggest that ecological complexity, tidal mixing, and dilution effects near the river mouth may contribute to increased community diversity. In contrast, lower diversity and evenness at W1 may result from limited nutrient inflow, upstream disturbances, or more homogeneous environmental conditions. Interestingly, W2 exhibited the lowest richness despite moderate Shannon diversity, possibly due to functional redundancy or the dominance of generalist taxa such as Microcystis under elevated nitrogen conditions. These patterns align with previous studies indicating that nitrogen enrichment and hydrological mixing are key drivers of microbial community structure in riverine systems [43,44]. Environmental filtering along the river’s longitudinal gradient may further select distinct microbial assemblages with site-specific adaptive traits [45,46].

### 4.3. Microbial Community Structure and Adaptation

At the genus level, Fragilaria, Microcystis, and Flavobacterium were the dominant taxa. Microcystis, a nitrogen-fixing cyanobacterium, was more abundant at W4 and W2 (W4: 15%, W2: 12%), consistent with high TN conditions (W4: 5.8 mg/L, W2: 3.5 mg/L), indicating its competitive advantage in nitrogen-rich environments. The high abundance of Microcystis may contribute to nitrogen replenishment through fixation, while its metabolites (e.g., microcystins) could suppress other taxa, altering community structure [47]. Flavobacterium, associated with organic matter degradation and nitrogen metabolism, was present across all sites (average abundance 8–10%), reflecting its broad adaptability to varying nitrogen levels. Fragilaria, a diatom, was more abundant at W3 (18%), likely due to the low-nitrogen (TN: 2.4 mg/L) and high-DO (9.0 mg/L) environment near the reservoir, which favors its growth. These results suggest that the Shi River microbial community adapts to varying nitrogen stress through adjustments in species composition and abundance, particularly exhibiting greater functional redundancy and competitive advantage at high-nitrogen sites like W4 [48].

Although eutrophication has not yet been directly observed in the Shi River study area, the microbial community analysis reveals a notable presence of genera adapted to nutrient-rich conditions, including Microcystis, Nitzschia, Cryptomonas, and Thalassiosira. These genera are known to thrive in environments with elevated nitrogen and phosphorus levels, such as those found in eutrophic lakes and reservoirs. Specifically, Microcystis, which dominated at sampling site W4 with a relative abundance of 15%, is a well-documented indicator of eutrophication due to its ability to form harmful algal blooms that can produce cyanotoxins, adversely affecting water quality and aquatic organisms [49]. Elevated water temperatures at W4, reaching 23–27 °C in summer (e.g., July 2022, Figure 2h), likely enhance the growth of Microcystis and other nutrient-adapted genera like Nitzschia and Thalassiosira as warmer conditions accelerate microbial metabolism and nutrient cycling, potentially exacerbating the risk of algal blooms [50]. Similarly, diatoms such as Nitzschia and Thalassiosira play significant roles in nutrient cycling by efficiently uptaking and transforming nitrogen and phosphorus, thereby influencing the overall nutrient dynamics of the river [51]. The presence of these genera suggests that the Shi River may be at risk of developing eutrophic conditions if nutrient inputs continue unabated. Warmer temperatures, particularly in summer, may further amplify this risk by promoting microbial activity and accelerating nutrient turnover, creating conditions more conducive to eutrophication.

### 4.4. Functional Genes and Responses to Nitrogen Availability

The Shi River microbial community exhibits dynamic responses to nitrogen availability, as evidenced by the spatial variation in functional gene expression and microbial community structure. Denitrification, a key process in the nitrogen cycle, appears to be more active at upstream sites (e.g., W1 and W2), where high nitrogen levels and relatively lower DO create favorable conditions for denitrifying bacteria [52]. The functional genes *narG* (encoding nitrate reductase), *nirS*, and *nirK* (encoding nitrite reductases) are expected to be more active in these nitrogen-rich, oxygen-limited environments, as denitrification is typically enhanced under such conditions [53]. This increased nitrogen availability, with NO_3_-N levels of several milligrams per liter (e.g., 2.1–2.8 mg/L at W1 and W2), likely promotes the functional diversity of denitrifying bacteria, such as members of the order Burkholderiales, by providing a sufficient nitrogen source for their metabolism. Although DO levels at W1 and W2 (7.5–8.5 mg/L) are relatively lower compared to W5 (9.5 mg/L), they are not hypoxic and still support denitrification without imposing constraints on microbial activity [54]. At downstream sites (e.g., W5), the lower nitrogen levels and higher DO suggest that denitrification is less active, which may explain the reduced activity of denitrification genes in these areas. However, if denitrification genes remain active at W5, other factors, such as microscale anoxic zones or alternative electron acceptors, may be influencing microbial activity, warranting further investigation.

Nitrogen assimilation, mediated by genes such as *glnA* (encoding glutamine synthetase) and *gdhA* (encoding glutamate dehydrogenase), appears to play a significant role across the Shi River, particularly in response to varying nitrogen availability. At upstream sites like W1, high ammonium and TN levels suggest that nitrogen assimilation is active, likely driven by the availability of ammonia, which is a substrate for *glnA* [55]. In contrast, at downstream sites like W5, the lower nitrogen levels indicate potential nitrogen limitation, which may upregulate *glnA* and *gdhA* to assimilate scarce ammonia resources [56]. At midstream sites (e.g., W2 and W3), moderate nitrogen levels suggest a balance between nitrogen assimilation and other processes, with *gdhA* likely contributing to ammonia incorporation into cellular metabolism [57]. These patterns highlight the microbial community’s ability to adapt to spatial gradients in nitrogen availability, optimizing gene expression to meet metabolic demands.

In contrast, the oxidative cycle of nitrogen, particularly nitrification, likely dominates at sites with higher DO levels, such as W3 (DO: 9.0 mg/L) and W5 (DO: 9.5 mg/L). Nitrification, the aerobic oxidation of ammonia to nitrate via nitrite, is driven by ammonia-oxidizing bacteria (AOB) and nitrite-oxidizing bacteria (NOB) through genes like amoA (encoding ammonia monooxygenase). At W3, the high DO and low TN (2.4 mg/L) favor nitrification, as evidenced by Limnohabitans’s contribution to complete nitrification (M00804, 20%) at this site. Similarly, W5’s high DO (9.5 mg/L) likely enhances nitrification, potentially upregulating *amoA* expression, as high oxygen availability supports ammonia oxidation [58]. Conversely, at W4, the lowest DO (6.8 mg/L) and high NH_3_-N (1.2 mg/L) may suppress nitrification, leading to ammonia accumulation. These patterns suggest that DO regulates the balance between oxidative (nitrification) and reductive (denitrification) processes in the Shi River, with nitrification prevailing in high-DO environments [59].

### 4.5. Functional Genes and Environmental Drivers of Nitrogen Cycling

Environmental factors significantly influenced nitrogen cycling in the Shi River by regulating functional gene expression. Mantel tests identified TN as the primary driver of community variation (r ≈ 0.6, *p* < 0.001), showing significant negative correlations with DO (r ≈ −0.6, *p* < 0.001) and pH (r ≈ −0.5, *p* < 0.001). This negative correlation was particularly evident at W4, where high TN (5.8 mg/L) and low DO (6.8 mg/L) likely promoted the expression of denitrification genes (e.g., K01915) while inhibiting nitrification [60,61]. DO was positively correlated with denitrification genes such as K00370 (narG) (r ≈ 0.6, *p* < 0.01), suggesting that higher DO enhances nitrate reductase activity, facilitating nitrogen removal [62]. Although denitrification is traditionally associated with anaerobic conditions, recent studies show that denitrification genes like *narG* can be expressed in oxic environments due to low-oxygen micro-niches (e.g., within aggregates or biofilms). These localized anaerobic zones allow denitrification to occur alongside aerobic processes, explaining the positive correlation between DO and *narG*. This reflects the spatial coupling of nitrification–denitrification and highlights the complexity of nitrogen cycling in river ecosystems [56]. However, NH_3_-N showed a negative correlation with these genes (r ≈ −0.55, *p* < 0.01), and high NH_3_-N levels (e.g., 1.2 mg/L at W4) may inhibit nitrogen metabolism enzymes, leading to nitrogen accumulation. K00760 (nifH, nitrogen fixation) exhibited increased activity in low-DO environments (e.g., W4) (r ≈ −0.5, *p* < 0.01), indicating that hypoxic conditions promote nitrogen fixation to replenish nitrogen sources [63]. These findings align with previous studies identifying DO and NH_3_-N as key regulators of nitrogen cycling. Additionally, agricultural runoff at W2 may further influence functional gene expression by increasing organic carbon (e.g., COD), warranting further investigation into the interaction between COD and nitrogen metabolism genes [64].

### 4.6. Microbial Interactions and Their Role in Nitrogen Metabolism

Co-occurrence network analysis of the Shi River microbial community revealed the intricate interplay between microbial genera and nitrogen metabolism genes, highlighting their roles in nitrogen cycling [65]. Key nodes include *g__Microcystis* (abundance = 2376) and *glnA*, *GLUL* (abundance = 24342), with *g__Microcystis* showing strong synergy with *narG*, *narZ*, *nxrA* (r = 0.9, *p* < 0.05) in nitrate reduction, but competition with *GLUD1_2*, *gdhA* (r = −0.9, *p* < 0.05) in glutamate metabolism. Similarly, *g__Limnohabitans* and *g__Reyranella* exhibit strong positive correlations with *glnA* and *GLUL* (r = 0.9–1.0, *p* < 0.05), suggesting functional complementarity in ammonia assimilation. Modularity analysis (Q ≈ 0.60) identified three functional modules: Module 1, centered on *glnA*, *GLUL* and *gltB*, supports ammonia assimilation; Module 2, led by *narG*, *narZ*, *nxrA*, drives nitrate reduction; and Module 3, involving *NRT*, *narK*, *nrtP*, *nasA*, facilitates nitrogen uptake. These findings align with recent studies on microbial nitrogen-cycling networks in urban rivers, which highlight cooperative dynamics under high nitrogen loads. The network’s functional redundancy, exemplified by *glnA*, GLUL’s multiple connections, enhances ecosystem resilience in polluted rivers, as noted in studies emphasizing the role of redundancy in maintaining microbial community stability under environmental stress [66]. Actinobacteria showed a negative correlation with Proteobacteria (r ≈ −0.5, *p* < 0.05), possibly reflecting resource competition (e.g., for nitrogen and carbon sources). Functional contribution analysis revealed that *unclassified_o_Burkholderiales* contributed the most to denitrification (M00529) at W4 (35%), while Limnohabitans contributed significantly to complete nitrification (M00804) at W3 (20%). The high variability in contributors to M00531 (assimilatory nitrate reduction) across sites likely reflects the spatial heterogeneity of environmental factors (e.g., DO and TN) influencing community function [67]. These results suggest that microbial communities regulate nitrogen cycling through both synergistic and competitive interactions, particularly exhibiting stronger functional synergy at high-nitrogen sites like W4 [68].

### 4.7. Future Research Perspectives

This study elucidated the drivers of nitrogen pollution, microbial community dynamics, and their roles in nitrogen cycling in the Shi River, providing a scientific basis for water quality management and pollution control. However, several unresolved questions warrant further investigation. First, the sources of nitrogen pollution in the Shi River remain incompletely characterized. Future studies could employ isotopic tracing techniques (e.g., δ15N and δ18O) to quantify the contributions of urban sewage and agricultural runoff, enabling more targeted pollution source control strategies [69]. Second, the regulatory mechanisms of DO and NH_3_-N on functional gene expression require deeper exploration. Transcriptomic and proteomic analyses could be used to investigate the transcriptional and translational changes of key genes (e.g., K01915, K00370), providing insights into their regulation under varying environmental conditions [70]. Additionally, the high abundance of Microcystis indicates a risk of eutrophication. To further our understanding of the microbial nitrogen cycle, we will include measurements of redox potential (ORP) in future studies. ORP can provide important insights into the direction of processes such as nitrification at high ORP (bbb100 mV) or denitrification at low ORP (<−100 mV) [71], strengthening the link between chemical parameters and microbial community composition. Future research should integrate long-term monitoring to assess the synergistic effects of nitrogen and phosphorus on algal blooms and develop microbe-based ecological restoration techniques (e.g., leveraging denitrifying bacteria for nitrogen removal) [72]. Finally, this study was based on data from 2021–2023. Expanding the temporal scale to analyze the long-term impacts of climate change (e.g., variations in rainfall and temperature) on nitrogen cycling and microbial dynamics could further enhance our understanding. These future investigations will provide a more comprehensive theoretical foundation for ecological restoration and water quality management in the Shi River.

## 5. Conclusions

This study elucidates the role of microbial communities in driving nitrogen cycling and contributing to pollution management in the Shi River by establishing a correlation between water quality, microbial diversity and functionality. The findings confirm substantial nitrogen pollution, with total nitrogen (TN) levels surpassing the 2.0 mg/L limit at several locations, especially during the rainy season, primarily due to agricultural runoff. The microbial community, predominantly comprising Fragilaria, Microcystis, and Flavobacterium, exhibited adaptability to elevated nitrogen conditions, as evidenced by the presence of functional genes such as *narG* (denitrification) and *nifH* (nitrogen fixation), which play pivotal roles in nitrogen transformation. Furthermore, microbial interactions have been demonstrated to influence nitrogen metabolism, as evidenced by co-occurrence networks that exhibited strong positive correlations between Proteobacteria and Bacteroidota, in contrast to competitive interactions with Actinobacteria. Functional analyses identified *unclassified_o_Burkholderiales* as a major contributor to denitrification at W4 and Limnohabitans to nitrification at W3, highlighting the community’s cooperative and competitive dynamics in nitrogen processing. The equilibrium between denitrification and nitrogen assimilation is influenced by nitrogen availability, dissolved oxygen, and organic matter, with upstream areas favouring denitrification and downstream regions leaning towards assimilation. These findings underscore the importance of integrating water quality, microbial diversity, and interaction data to elucidate nitrogen cycling in river systems, providing a foundation for precise pollution control measures. Future research should investigate factors such as temperature, salinity, and small-scale environmental variations to better understand microbial activity drivers in the Shi River, especially where gene expression diverges from expected environmental patterns.

## Figures and Tables

**Table 1:** Analysis of alpha diversity.

Sample	Ace	Chao	Shannon	Simpson	Shannon’s Evenness
W1	5776	5776	4.643351	0.041469	0.536093
W2	5119	5119	5.032051	0.023646	0.589184
W3	5702	5702	4.877654	0.0244	0.563984
W4	5986	5986	5.170575	0.019274	0.594512
W5	5881	5881	5.252409	0.014341	0.605152

## Data Availability

The datasets generated and/or analyzed during the current study are available from the corresponding author on request.
